# Women’s experience of psychological birth trauma in China: a qualitative study

**DOI:** 10.1186/s12884-020-03342-8

**Published:** 2020-10-27

**Authors:** Ke Zhang, Ling Dai, Meiliyang Wu, Tieying Zeng, Mengmei Yuan, Ye Chen

**Affiliations:** 1 Department of Nursing, Tongji Hospital, Tongji Medical College, Huazhong University of Science and Technology, 1095 Jiefang Avenue, Wuhan, 430030 China; 2 School of Nursing, Tongji Medical College, Huazhong University of Science and Technology, 13 Hangkong Road, Wuhan, 430030 China

**Keywords:** Psychological, Birth trauma, Experience, Women, Childbirth, Qualitative research

## Abstract

**Background:**

The psychological birth trauma is a universal phenomenon in childbearing women. The influences could extend in a wide range, which includes the mothers’ health, mother-infant relationship, partner relationship. The medical staff could even choose to quit playing their part in the birthing process. The phenomenon has gradually garnered attention around the world. However, it has rarely been discussed under Chinese special conditions. The study was to explore Chinese women’s lived experiences of psychological birth trauma during labor and birth.

**Methods:**

A descriptive phenomenological approach was adopted in this study. Twenty-four women were recruited, who reported having experienced psychological birth trauma. In-depth interviews were conducted within 1 week after birth. Colaizzi’s method was used to analyze the data.

**Results:**

Twenty-four women participated in the study. Four themes emerged to describe the women’s experience of psychological birth trauma: “How am I supposed to relieve the endless pain?” “ Can’t I be weak?” “Am I not important?” “What uncertainties are waiting for me?”

**Conclusions:**

The findings provide deep insight into Chinese women’s unique experience of psychological birth trauma. The social and health system could prevent psychological harm during birth and promote maternal health by measures of pain management, thoughtful attention, adequate caring, and prenatal preparation.

**Supplementary information:**

The online version contains supplementary material available at 10.1186/s12884-020-03342-8.

## Background

Birthing a child is an endowed privilege that only a human with a uterus can have. However, most of the time, this process is accompanied by excruciating pain, and occasionally by physical and psychological trauma [1]. With modern surgical techniques, the wounds of physical trauma usually heal, while the wounds of psychological trauma constantly linger [2]. Psychological birth trauma, also known as traumatic childbirth, refers to the maternal severe psychological harm caused by the events occurring during labor and birth [3, 4].

Psychological trauma caused by childbirth is a universal phenomenon and has an extensive effect. The global prevalence of traumatic childbirth experience is estimated between 9 and 44% [2]. The harms of psychological birth trauma might affect the mothers’ health, mother-infant relationship, partnership, and even spread to medical staff [5]. As for women themselves, psychological birth trauma may lead to post-traumatic stress, which is characterized by a traumatic memory, negative cognition, and escape behavior, in severe cases, even progresses to posttraumatic stress disorder or suicide [6, 7]. In terms of the mother-baby relationship, the infants may not be breastfed as a result of mothers’ estrangement and disgust [8]. Because of women’s misery from pregnancy to childbirth, the husbands may blame themselves, or feel dreadful and helpless, which leads to marital conflict and strain [2, 9]. In the case of medical staff, some midwives and nurses may experience secondary traumatic stress after witnessing the childbirth process with sympathy and empathy to the mothers [10].

The potential causes of women’s traumatic experience during childbirth vary from individual, fetal, and social factors. Firstly, the individual factors include the history of psychotic disorders, history of sexual abuse, prenatal depression, previous trauma, and high expectation, all of which may conspire to spawn negative cognition among postpartum women [11–13]. Secondly, premature or unhealthy newborns could deal with a traumatic blow to the women, who are usually plunged into endless anxiety and worry [14]. Thirdly, women might feel neglected and abandoned due to the lack of good care, poor social support, or ineffective communication with medical staff and family [15, 16].

Professor Beck was the first scholar to look into psychological birth trauma, for which she summed up five attributes, including being deprived of caring, being stripped of their dignity, terrifying loss of control, neglected communication, and being buried and forgotten [5]. Later on, the issue has garnered international concerns, and researches into this field began to sprout sporadically in some countries. An Irish study about the experience of traumatic childbirth found that the women reported being dismissed, dehumanized, and passive during childbirth [17]. One qualitative research gained the perception of traumatic childbirth from Iranian mothers, who described childbirth suffering as apprehension, helplessness, and a sense of impending death [3]. A Spanish study found that a majority of responses from women living a traumatic childbirth experience mentioned feelings of being un/misinformed by healthcare personnel, being disrespected and objectified, lack of support, and various problems during childbirth and postpartum [18].

There is a paucity in the evidence about the traumatic experience during labor and birth for Chinese women. China has a unique social environment and cultural background. In recent years, the Chinese new policy allows couples to have two children, which has seen a spike in the number of multiparas, especially those with advanced maternal age. In China, the number of births was at the level of 14.65 million in 2019 [19]. This means the large population of Chinese women may risk the possibility of experiencing psychological birth trauma. How Chinese women feel about traumatic childbirth remains unclear. The research about Chinese women could enrich the connotation of psychological birth trauma on current evidence. Therefore, this research aims to explore women’s experience of psychological birth trauma in China.

## Methods

### Study design

The study used a descriptive phenomenological qualitative approach by in-depth interviews with women after childbirth. The descriptive phenomenology was set up by Husserl. He claimed that the source of knowledge was the phenomena themselves, and emphasized describing the experience without explanation [20]. This method can help to reveal the traumatic experience during labor and birth, and better understand and grasp the essence of psychological birth trauma. The reporting followed the consolidated criteria for reporting qualitative research.

### Participants

Purposive sampling was adopted to recruit research participants from obstetric wards of a general hospital, Wuhan, China. The study mainly focused on the particular characteristics of women who had experienced psychological birth trauma. The inclusion criteria included: (1) women who self-reported they had experienced traumatic childbirth, (2) more than 18 years old, (3) within the first week after delivery, and (4) signed informed consent form to take part in the research. The sample size was determined based on sampling saturation, which meant no further sampling was necessary [21]. The final interview was conducted with an additional participant until there is no new theme. Meanwhile, the research tried to make sure of the biggest differences between the participants. Two participants were excluded because unstable conditions interrupted the communication. Finally, 24 women were recruited in the study.

### Data collection

Data were collected by face-to-face, semi-structured interviews from May to August 2018. The interviews lasted 43–90 min for each woman. The interviewers introduced the meaning of traumatic childbirth to make sure the women understand, then asked, “Did you have a traumatic childbirth experience?”. If the answer was yes, we deemed that she self-reported psychological birth trauma. If she met the other inclusion criteria, we could consider inviting her to participate in the research. The interview questions were as follows: “Could you please describe the experience of psychological birth trauma in detail?”. “Would you share any other information that has not been mentioned?” (See supplementary file 1). In this research, six female researchers, among whom two had a doctoral degree in nursing, one had a master’s degree in nursing, and three were the doctoral degree candidates of nursing. All interviewers had been trained for interview skills before the interviews. To make sure no distraction during the interviews, the researchers made an appointment with the participants and prepared a quiet office without other people present. The researchers were permitted to record by an audio device the whole interview and write the interview content. In addition, the researchers took note of the participants’ mood, facial expression, and body language wherever we deemed necessary during the interview.

### Data analysis

To ensure the accuracy of data, the researchers listened to the audio recordings repeatedly. The researcher transcribed the audio recordings in words within 24 h after each interview and added the notes of the interview details. The transcripts were returned to participants for comment and correction. We gave every participant an ID as the pseudonym to protect their privacy. Two researchers processed the data by Colaizzi’s method, which was commonly applied in phenomenological research. The process included seven steps: transcribing the data in detail, excerpting the significant statements, concluding and extracting the meaning, finding the characteristics and forming themes, developing the intact description, stating the essential structure of the phenomenon, and proving the truth of the content [22].

### Ethical consideration

Ethical approval was obtained from the Ethics Committee of Tongji Hospital of Tongji Medical College of Huazhong University of Science and Technology. The investigation gained the permission of chief nurses who managed the regular work of the obstetric wards. Before interviewing, all researchers had no prior relationship with the participants. The researchers introduced themselves and explained the aims and methods of the study. All research participants signed informed consent. The participants were given the option to withdraw from the research process at any time. And the researchers promised not to use any personal identifier in research reports or publications. When the participants expressed stress apropos of recalling the trauma experience, the researchers would pause to comfort the participants until they could carry on. The interviews followed the principles of information privacy and concealment.

## Results

In total, 24 women participated in this study. The median age of the participants was 32 years (Interquartile range, IQR: 29–34.25 years). The median gestational age was 36.5 weeks (IQR: 34.5–39.4 weeks). Table 1 shows more general information.

**Table 1:** Demographic characteristics of participants (*N* = 24)

Demographic characteristic	N	%
Parity		
Primipara	13	54.2
Multipara	11	45.8
Education level		
Undergraduate	16	66.7
Middle school	7	29.2
Primary school	1	4.1
Occupation		
Yes	18	75.0
No	6	25.0
Planned pregnancy		
Yes	19	79.2
No	5	20.8
Mode of birth		
Natural childbirth	9	37.5
Inducing labor	2	8.3
Planned cesarean section	10	41.7
Emergency cesarean section	3	12.5

Through Colaizzi’s 7-step approach, four themes and ten subthemes were extracted from the women’s experience of psychological birth trauma, which was illustrated in Table 2.

**Table 2:** Identified themes and subthemes

Themes	Subthemes
How am I supposed to relieve the endless pain?	Excruciating pain
	Inaccessibility to labor analgesia
Can’t I be weak?	Constant pressure
	Burden of expectation
Am I not important?	Powerlessness to decide
	Gender preference
	Neglect by the medical staff
What uncertainties are waiting for me?	Unknown process
	Security concern
	Tense atmosphere

### How am I supposed to relieve the endless pain?

There was no doubt that the unbearable pain during childbirth was one of the most unforgettable memories for women. Although the expectant mothers knew the pain was inevitable, the intensity and endurance of the pain were far beyond what they had imagined. The fear of labor pain and loss of self-control became the leading causes of psychological trauma for childbirth. When the pain management was failing, the endless physical pain would deal a psychological blow, causing terrible suffering to the women.

#### Excruciating pain

The physical pain appeared throughout all the stages of labor and could inevitably lead to psychological distress. At the first stage of labor, the paroxysmal uterine contraction contributed to intense abdominal pain. At the second stage of labor, the mother needed to push as hard as they could to help to accelerate the birth process. The injury of the birth canal and the laceration of the perineum might cause great pain to the perineum during the process. The labor pain was far beyond what the women could bear and began to crush them.

*"In the beginning, I could tolerate the pain. I even had some light sleep until noon. Then the pain intensified around 3 o'clock that afternoon. I felt like I was near death because of the pain. It could not be relieved, no matter what you do." (P18)*

*"The fetal biparietal diameter is longer than the others. The birthing process was accompanied by piercing pain when the baby came out. At that moment, I felt myself being torn apart. In general, I don't like to cry even if I am in pain, as I practiced yoga before. I knew how to relieve pain by breathing training. But this time, it made me break down during birthing. I yelled and cried out." (P10)*

#### Inaccessibility to labor analgesia

Labor analgesia could help expectant mothers to relieve labor pain, which includes non-medical methods, medical methods, and epidural anesthesia. But the technique has not been widely applied to women in China. Many hospitals do not provide the service of labor analgesia for women, mainly as anesthetists are understaffed. Therefore, the women, especially those who experienced natural childbirth or emergency cesarean section, had to bear the endless excruciating labor pain. The pain could not be effectively eased and might drive them mad.

*"I chose this hospital because I had known it could provide the service of painless childbirth. But when I was moved to the birth room, the doctor told me that the hospital had only one anesthetist who had the expertise of labor analgesia, and the very one was not available at the moment due to a business trip. I could not believe my ears when I heard it. The pain was so excruciating that I wanted to knock my head against the wall. Nobody had informed me that only one doctor could practice the technique in such a general hospital and that he was not on duty." (P8)*

### Can’t I be weak?

The emotional vulnerability which resulted from heavy ideological baggage was considered as a psychological trauma for expectant mothers. It is generally known that childbirth is one of the most natural processes in the human world. And nothing can be more natural than vaginal birth to have a child. Furthermore, there is a widely acclaimed notion in China that one woman should be mentally strong once she becomes a mother. The conventional perspective is widespread both in the general population and among healthcare workers caring for these women. All people emphasized the point, but it seemed to take no count of the childbearing women’s psychological states.

#### Constant pressure

The families were repeatedly vocal of the fact that every woman had to go through all the sufferings to give birth to a baby, and that over the course of human history, all women have their babies with the same suffering. Thus, the expectant mothers had sort of frustration when they didn’t feel heard and understood.

*"Everyone said that it was not easy to give birth to a baby. My family kept telling me that some women spent two or three days to deliver the baby; some even took one week and that every pregnant woman had to endure the same pain to become a mother. You must bear severe pain on the body and mind throughout labor and birth. Such a saying may go well with others, but definitely not me. I don't wanna be like others. They all get me wrong, and nobody could understand my suffering. The birthing process was utterly physical and mental torture." (P3)*

#### Burden of expectation

It is widely recognized by the Chinese that once a woman becomes a mother, she ought to reshape herself into a figure with great fortitude, perseverance, and resilience in order to go through all the difficulties and the hardship that await her ahead. Actually, the expectant mothers might not have a heart as strong as other people think they ought to have. In their opinion, a strong heart didn’t fall in their lap like pennies from heaven, and they found it difficult to come up with such anticipation, which destroyed their mind. The repetition of the stereotype even made them felt upset and fragile.

*"The doctor emphasized it is impossible for women to give birth with no pain over and over again. I was more disappointed when I heard that. Certainly, I did know childbirth came along with severe pain. I felt great pain already. Why did he still underscore the point? At least he should have given me some encouragement and confidence. In that situation, as a patient, the pain had maxed me out. I was fragile and didn't hope to hear the negative words." (P8)*

### Am I not important?

When going through the birthing process, the women usually thought they did not receive enough attention and support from family and medical staff. Family support, as one part of social support, played an important role in stabilizing emotions. However, the family members always insisted on what they thought was right rather than the women’s inner demands. The women’s decisions on their body and delivery mode were ignored by their families. Moreover, the medicals were busy providing therapeutic services to expectant mothers, but they could not focus their mind on a certain woman during work time. Childbearing women felt neglected and forgotten by relatives and professionals, which gave rise to the negative experience.

#### Powerlessness to decide

The women did not feel empowered to decide on their bodies and delivery mode. The family members, who were deeply convinced by the advantages of natural birth, did not respect the choices and decisions of the childbearing women. When the women in great pain begged for a cesarean section, the family refused the request and urged the women to hold onto natural childbirth. Such a rejection made the women sad.

*" How I wish to die! The terrible pain continued for six hours. I didn't want a natural birth. I wanted to have a C-section. But my mother strongly expected me to give natural birth. She even lied to me that the doctor was absent." (P19)*

*"At that time, I felt abandoned by the world. Nobody cared about my pain and understood me. They all thought I was effeminate. I denied the words. I felt so painful, but my mother couldn't understand. How could she say that?" (P22)*

#### Gender preference

When suffering the physical and mental torture of birth, the women were easier to feel neglected by their families, especially their husbands, as they did hope their families to take more care of them. Affected by the deeply-rooted prejudice that only sons could carry on the family line, some families were eagerly looked forward to having a male heir. What they only cared about was the baby’s gender, dismissive of the women’s feelings and thoughts.

*"This pregnancy was unplanned. I didn't want another child. I wanted to be free. But my family greatly hoped to have a boy to carry on the family name. They insisted that you won't be laughed at until you have a son." (P2)*

*"After surgery, I was left alone approximately for 20 minutes at the door of the operating room. I didn't know where my husband and mother were. I felt angry because they were chatting when the doctor tried to reach my family." (P12)*

#### Neglect by the medical staff

The medical staff’s every move was watched and affected the childbearing women during birth. The women thought they deserved the accompany and encouragement from the medical workers. But in many circumstances, medical workers were busy getting their job done. When lacking enough care from doctors, nurses, or midwives, women would lose confidence and feel being left in the lurch without any psychological support.

*"The medical staff left me hanging. They changed shift at eight in the morning. The handover meeting took more than one hour. My call for them was met with ignorance. I felt like being abandoned." (P6)*

### What uncertainties are waiting for me?

The women would be faced with many uncertain situations during labor and birth. The fear of unknowns made them regard childbirth as a disaster. Meanwhile, a multitude of objective factors could not be predicted before birth, and emergencies were common during birth. Childbearing mothers usually worried about terrible things that could go wrong and lost their confidence in handling unexpected conditions.

#### Unknown process

The expectant mothers could not independently decide the mode of birth as they originally planned. A medical emergency could happen during the birthing process as a result of antenatal objective factors, as in the abnormal positioning, or cesarean history, etc. On some occasions, the expectant mothers had no choice but to shift to a cesarean section from a failed vaginal birth attempt, though they had strained themselves through the long hours and the tormenting pain. These women felt hopeless as they had to sustain dual pain, including from a failed natural childbirth attempt and from a cesarean section.

*"I was almost collapsed at that moment. The birthing process was awfully lengthy, and I still failed to push my baby out. The unknown situation plunged me into terrible fear. I dared not risk my baby's life and had to give up. It compelled me to resort to C-section at last. I suffered a lot. I felt squashed and cried inside (the operating room)." (P3)*

#### Security concern

During the whole labor process, the expectant mothers usually felt apprehensive because of perceived dangers threatening themselves or babies. They worried that their underlying diseases or low labor force might become an obstacle to a successful birth. Besides, the women always considered fetal safety first so that the preterm birth, fetal distress, and other unfortunate situations also made them full of fear.

*"The surgical procedure took a long time. I lay on the bed uncomfortably. The doctor told me that my bladder adhesion was quite severe due to the previous cesarean section. I lost my composure all of a sudden. I felt scared and nervous because I didn't know what that meant to me." (P11)*

*"I was not in the hospital when the amniotic fluid leaked. I couldn't calm down and was panicky. It frightened me that the baby appeared to rush out so early. I worried that my baby didn't fully grow. Besides, I didn't know how to make the choice of cesarean section or natural birth." (P10)*

#### Tense atmosphere

Under the influence of other expectant mothers, the women were easier to feel stressed and fearful. When waiting in the labor room, the women witnessed other expectant mothers’ negative experiences and emergency circumstances. They were afraid that the same thing might happen to themselves and had no control over the process, which rendered them into emotional incontinence.

*"I felt horrible when witnessing the childbirth process of other women. Firstly, I found that I was older than these first-time mothers. Moreover, I had my first baby by cesarean section, which resulted in the scarred uterus. When I saw they had a rough time, I felt more scared." (P22)*

*"The process of cervical dilation was like a nightmare. I still feel scared up to now. Especially, the mothers shared the same room. When they yelled during cervical dilation, I felt petrified. I didn't know how long the condition would last and how serious the pain would be." (P6)*

## Discussion

As the first study on psychological birth trauma for women in mainland China, the researchers conducted qualitative interviews with women within the first week after birth to focus on the women’s experience of traumatic childbirth. After the analysis of collected data, four themes and ten subthemes emerged in the context of Chinese social culture. “How am I supposed to relieve the endless pain?” “Can’t I be weak?” “Am I not important?” “What uncertainties are waiting for me?” Based on the gathered information, the research found that the women who experienced traumatic childbirth had a similar experience. They described the psychological birth trauma as psychological distress due to unbearable physical pain, emotional vulnerability under a heavy mental burden, feeling neglected, and fear of uncertainties. The details of the results in these areas would then be discussed.

This study found that the psychological suffering of women caused by severe physical pain was a prominent manifestation among women who reported childbirth trauma, which was aligned with the existing evidence from Ireland and Spain [17, 18]. Labor analgesia was an effective way to manage labor pain. It could reduce the risk of postpartum psychological disorders of women [23]. However, labor analgesia was not widely used in China [24]. Especially in general hospitals, the shortage of anesthetists renders plenty of women unable to receive the service of analgesia during childbirth [25]. Under this circumstance, the National Health Commission of the People’s Republic of China rolled out a pilot project of labor analgesia in 2018, which aimed to achieve safe birth and promote the mental health of birthing women [26]. At present, labor analgesia is still at an early stage in China and being pushed forward rapidly [27]. Besides, the practical approach is to form the specialized team to offer the service of labor analgesia in the whole process of childbirth, and help the women to draw up a plan for managing labor pain. It is particularly urgent to make labor pain management accessible to more childbearing women, as many of them experience a traumatic birth physically and psychologically.

In this research, the traditional views brought tremendous pressure to the childbearing women and made them experience traumatic childbirth. On the one hand, most people took for granted that it was a natural process for women to give birth [28]. On the other hand, the situation was common in China that the women were expected to have a strong mind for their upcoming babies. This expectation has resulted in a heavy mental burden that could consume women’s energy and therefore becomes a trigger to psychological birth trauma. However, the issue of traumatic childbirth has not been given as much attention as postnatal depression, fear of childbirth [29, 30]. It is important to promote greater understanding and provide more encouragement of each woman to give birth naturally, especially those that had a high-stress level [31]. Reducing the psychological burden of childbearing women might be helpful to keep them away from the psychological trauma of childbirth.

The participants expressed that the family and medical staff neglected their feelings and needs. This finding falls in line with the results from Beck and Almagro [16, 18]. The majority of women were willing to give birth to a new life for their families despite the high risk. In return, support from family members was crucial, especially from their partners. Partner support was a protective factor for the perinatal psychological health of the women, which could reduce the negative maternal evaluation of childbirth [32, 33]. On the other hand, the women expressed the need for professional medical support, which was supported by a previous study [34]. The support from medical staff could reduce the negative psychological experience of birth [35], improve maternal and neonatal outcomes [36], promote the adaptation of maternal role, and increase maternal satisfaction and self-confidence [37]. Therefore, it is necessary to beef up the support from not only the family but also from medical staff so that the childbearing women could get more attention and avoid psychological birth trauma.

The Iranian women were reported the fear and anxiety that affected them from having another pregnancy in the future [3]. Our findings showed that the expectant mothers feared the uncertainties during birth, especially premature birth and newborn health problem, which was a supplement for the connotation of fear. It is a feasible way to reduce uncertainties during childbirth by tracing the prenatal factors. Therefore, the medical staff could provide professional help to childbearing women before birth, which could reduce the fear of unexpected uncertainties and avoid traumatic experience during childbirth [38]. As an indispensable part of prenatal care services, prenatal education could contribute to reducing the fear of childbirth and improving maternal and infant health [39]. The contents could cover health in pregnancy, the birthing process, and postnatal family planning. For the preparation of birth, nurses could help expectant mothers make a birth plan and give more explanation about the early signs and symptoms of labor, the mechanisms of labor, the techniques of pain management during birth, and the likes [39, 40]. Full preparation would increase the confidence of women during childbirth, and make them concentrate on personal conditions without being swayed by others. Thereby, the women would ward off traumatic childbirth.

### Limitations

Though the number of participants in this research ensured data saturation, these women might only reflect the characteristics of psychological birth trauma in a specific geographic location of China. In some rural areas, it might be harder for birthing women to receive labor analgesia, which could increase the risk of a traumatic experience. Hence, further study could recruit more women from different geographical locations within mainland China to enrich the connotation of traumatic childbirth and explore the influencing factors of traumatic childbirth. On the other hand, the study recruited women who self-reported psychological birth trauma, without an objective screening. An instrument measuring psychological birth trauma is in need of development to confirmed psychological birth trauma for women in future researches.

## Conclusions

The research explores Chinese women’s experience of psychological birth trauma. The findings provide deep insight into the women’s feelings and mental needs during labor and birth. They suffer mental torment of pain, the psychological burden of stereotypes, being neglected by family and medical staff, and fear of the unknown during birth. To reduce psychological birth trauma and promote maternal and infant health, the research suggests taking measures about pain management, thoughtful attention, adequate caring, and prenatal preparation.

## Supplementary information

**Additional file 1.** Interview guide

## Data Availability

The datasets used and analyzed in this study are available from the corresponding author on reasonable request.

## Abbreviation

IQR: Interquartile range
