# Supplementary material for: Women’s experience of psychological birth trauma in China: a qualitative study
Source: BMC Pregnancy Childbirth. 2020 Oct 27;20:651. doi: 10.1186/s12884-020-03342-8 (PMC7590597; doi:10.1186/s12884-020-03342-8)
Supplement: Supplementary file 1 — Additional file 1. Interview guide [file 12884_2020_3342_MOESM1_ESM.docx]

**Interview guide**

Interviewee Number： Interview date：

Interviewer： Duration of interview:

**Background information:**

Age: Education level: Occupation: Parity: Planned pregnancy: Mode of birth:

**Interview questions:**

1. Which things made you feel bad during labor and birth? Did these things cause psychological trauma to an extent?
2. Could you please describe the experience of psychological birth trauma in detail?
3. How did you cope with them?
4. What can hospitals or medical staff do for you or other women with similar experiences to reduce psychological birth trauma?
5. Would you share any other information that has not been mentioned?
